# Hypermentalizing: the development and validation of a self-report measure

**DOI:** 10.3389/fpsyg.2025.1546464

**Published:** 2025-07-04

**Authors:** Carla Sharp, C. Barr, Salome Vanwoerden

**Affiliations:** ^1^Department of Psychology, University of Houston, Houston, TX, United States; ^2^STEM Programs, Rice University, Houston, TX, United States; ^3^Department of Psychiatry, University of Pittsburgh, Pittsburgh, PA, United States

**Keywords:** hypermentaling, personality disorder, assessment, psychopathology, theory of mind

## Abstract

**Introduction:**

Hypermentalizing (referred to as excessive theory of mind or biased mindreading) is defined as the tendency to make assumptions about other people's mental states that go beyond observable data. Despite recent interest in this construct, no self-report measure of hypermentalizing exists. The aim of the current study was to fully operationalize the construct of hypermentalizing by developing a theoretically grounded (attachment-based) self-report measure of hypermentalizing assessing mentalizing related to parents, peers and intimate partners; and evaluate the new measure for its psychometric properties.

**Methods:**

In Study 1,745 undergraduate students (mean age 21.12; *SD* = 2.19) completed the Hypermentalizing Questionnaire (HMZQ) alongside an experimental measure of mentalizing (the Movie Assessment for Social Cognition; MASC).

**Results:**

Results of factor analyses with MASC scores for external validity confirmed the purported factor structure of the HMZQ and suggested superiority for the HMZQ version that assesses mentalizing in relation to parents. Study 2 compared HMZQ scores in 364 adolescents between 12 and 17 years of age (70 adolescents with BPD, 136 psychiatric controls, and 158 healthy controls), and confirmed the superiority of the 26-item version of the HMZQ that assesses mentalizing in relation to parents, in that it was only the HMZQ version that distinguished borderline personality disorder from other psychiatric disorders and healthy controls.

**Discussion:**

The current study provides evidence in support of the HMZQ to assess hypermentalizing in typical and atypical populations of adolescents and young adults.

## Introduction

Mentalizing is a multi-component construct defined as the capacity to reflect on one's own thoughts and feelings and those of others to predict and understand behavior in the context of interpersonal interactions and relationships (Bateman and Fonagy, 2012a,b). The concept has been used in psychoanalytic literature since the 1970s (Allen, 2003; Marty, 1991; Marty and M'Uzan, 1963) to refer to the process of mental elaboration, including symbolization for the transformation and elaboration of drive-affect experiences as mental phenomena and structures (Lecours and Bouchard, 1997). It was incorporated into mainstream neurobiological and developmental literature (Frith, 1992; Morton, 1989) in the 1980s and 1990s, where it has been used interchangeably with the more frequently used concept of “theory of mind” (ToM). Premack and Woodruff (1978) coined the term “theory of mind” to refer to the capacity to interpret other people's behavior within a mentalistic framework in order to understand how self and others think, feel, perceive, imagine, react, attribute, infer, and so on. Mentalizing lies at the very core of our humanity because without the capacity to reflect on our own and other's mental states, we cannot maintain constructive social interaction, mutuality in relationships, or a robust and integrated sense of self (Bateman and Fonagy, 2012a,b).

The capacity to mentalize is theorized to develop within the context of secure attachment relationships with primary caregivers (Fonagy et al., 2002). Empirical studies support this notion with prospective studies having demonstrated that secure attachment facilitates the development of mentalizing (Fonagy et al., 1991; Meins, 1998; Symons and Clark, 2000). Conversely, disruptions in attachment relationships are associated with impaired mentalizing capacity, both prospectively (Belsky et al., 2012) and cross-sectionally (Sharp et al., 2015b,a; Ensink et al., 2015). In turn, impairment in mentalizing has been demonstrated for almost all types of psychopathology in youth (see Sharp and Venta, 2012 for a review) and adults (see Brune and Brune-Cohrs, 2006 for a review). Emerging from this literature are two broad types of mentalizing impairment: hypomentalizing and hypermentalizing (Abu-Akel, 2008; Crespi and Badcock, 2008; Fonagy et al., 2016; Gambin et al., 2015). *Hypomentalizing* reflects a deficiency in (lack of) mentalizing; that is, an inability to consider complex models of one's own mind and/or that of others (Fonagy et al., 2016). This deficiency is likely due to a reduced capacity to attribute thoughts, feelings and intentions (i.e., mental states) to oneself and others, resulting in comprised ability to make sense of social cues and interpersonal interactions. A large body of literature demonstrates an association between hypomentalizing and a wide variety of disorders, including autism (e.g., Baron-Cohen, 2000), psychopathy (e.g., Sharp et al., 2015a), and conduct problems (e.g., Happé and Frith, 1996; Sharp, 2008).

In contrast, *hypermentalizing*, which has also been referred to as excessive theory of mind (Dziobek et al., 2006) or biased mindreading (Sharp, 2000), involves making assumptions about other people's mental states that go beyond observable data (Crespi and Badcock, 2008; Fonagy et al., 2016; Gambin et al., 2015; Sharp, 2014; Sharp et al., 2011; Sharp and Vanwoerden, 2015). As such, it involves over-attribution of mental states and intentions to others, their likely misinterpretation, and the urge to act in response to the assumed mental states of others. It furthermore involves the over-interpretation of one's own mental states and a conflation of self-other mental states (Frick et al., 2012) or overactive and exaggerated resonance with the mental states of others due to confusion between self-and other-mental states (Ensink et al., 2015; Sharp and Vanwoerden, 2015). Hypermentalizing is by its very nature indicative of a metacognitive deficit since it involves failure to attain a higher-order representation from which to question one's own belief in service of generating alternative hypotheses in interpreting situations about the self and others (Semerari et al., 2005, 2007). As such, hypermentalizing reflects a lack of metacognitive differentiation (Semerari et al., 2005), because representation is conflated with reality. In summary then, hypomentalizing represents deficient (under) use of mental states in explaining behavior in self and others, while hypermentalizing represents over-use of mental states in making sense of self and others. In contrast, optimal mentalizing entails the use of mental states to understand self and others in productive ways. For instance, the optimal mentalizer would use mental states to explain behavior, but would do so from a stance of curiosity, openness and flexibility. The optimal mentalizer would ask whether a feeling, thought or intention is associated with behavior, but would not assume such mental states. Finally, the optimal mentalizer is able to flexibly integrate new information as a representation of another's (or own) mind is constructed in service of understanding and explaining behavior (Sharp and Bevington, 2022).

Compared to the evidence base on hypomentalizing in psychopathology, the hypermentalizing literature is much smaller, which is partly due to a lack of measures to assess the construct. Hypermentalizing first appeared in the literature in the context of schizophrenia (Langdon and Coltheart, 1999; Langdon et al., 2006a,b). Patients with schizophrenia have been found to overattribute intentions; misplace emphasis on stimuli thereby prompting inferences of abnormal meaning, see patterns that other people do not perceive, draw conclusions on less information, and report false-positives in ambiguous situations (Abu-Akel and Shamay-Tsoory, 2011; Grant et al., 2014; Howes and Kapur, 2009). More recently, hypermentalizing is also assessed as a key feature in Borderline Personality Disorder (BPD; Bo et al., 2015; Franzen et al., 2011; Frick et al., 2012; Preissler et al., 2012; Sharp et al., 2011), and can be detected most clearly in long and overly detailed accounts that have little or no relationship to reality, coupled with inflexible certainty in beliefs about others' mental states (Fonagy et al., 2016).

The increase in interest in the construct of hypermentalizing calls for the development of reliable and valid tools for its assessment. The most commonly used tool is the Movie Assessment for Social Cognition (MASC; Dziobek et al., 2006) which is an experimental task in which research participants are presented with four mutually exclusive options in response to a video clip of interaction partners: hypermentalizing, hypomentalizing, no mentalizing and accurate mentalizing. While this task has been shown to be valid and reliable in many studies across various populations, it is time consuming and can take up to 45 min to complete. As yet, a relatively quick and easy-to-administer self-report tool for the assessment of hypermentalizing is lacking. Apart from the practical advantages associated with the brevity and ease of administration of self-report, an additional advantage relates to the fact that the MASC is a performance-based measure. It is well-known that performance-based measures tap into one aspect of a construct while self-report measures tap into the more conscious, representational aspects of the construct. Both are important and provide important insight into mentalizing through different lenses.

The aim of the current study was to develop a self-report measure of hypermentalizing and evaluate the newly developed measure for its psychometric properties. In the development and evaluation of the measure, a few considerations were taken into account. First, a review of the hypermentalizing literature revealed that hypermentalizing contains five elements (Sharp, 2014; Sharp and Vanwoerden, 2015): an overconcern with the mental states of others; overinterpretation of others' mental states; inflexible certainty in own beliefs about others' mental states; acting impulsively on the assumed mental states of others; and second-guessing or over-interpretation of own mental states. These related components were identified as forming potential subscales. Items were subsequently written for each.

Second, given that the concept of mentalizing, at least in the context of personality disorder, has its roots in attachment, our intention was to develop a measure of hypermentalizing that acknowledges this theoretical basis. Specifically, Fonagy and colleagues' model for the development of personality disorder suggest that it is through secure attachment with caregivers and its associated parental reflective functioning that the mentalizing capacity of the child emerges (Fonagy et al., 2002; Sharp and Fonagy, 2008). In short, infants and young children do not yet have the reflective capacity to help them make sense of self and others. The development of mentalizing capacity therefore relies on a process called “marked mirroring” by which caregivers mark their offspring's internal experiences and give it back to the offspring in digested form. In this way, over time, and as the child's own reflective capacities increase, the infant/child comes to know their own mental states and develop a capacity for reading the mental states of others. If caregivers are intrusive in their marked mirroring or passive, or inconsistent, or non-contingent, atypical mentalizing styles, including hypo-and hypermentalizing develop (see e.g., Kim, 2015 for a review). Against the background of this theoretical and empirical evidence, we deemed it important to relate items of the newly developed mentalizing measure directly to individuals' attachment context. Participants were therefore asked to answer questions about thoughts and feelings that are typical for them in interaction with significant others. Relatedly, against the background of research showing attachment and mentalizing is relationship-specific (O'Connor and Hirsch, 1999), it was important that the measure be sensitive to the specific attachment context. Therefore, questions were asked three times over: in relation to parents, romantic partners and closest friend. These three attachment contexts were chosen because of their unique relevance for the developmental period of adolescence and young adulthood. Specifically, research has shown that adolescents typically increase their valuation of peer and romantic partner relationships, develop greater psychological distance from parents, and renegotiate boundaries and responsibilities in family relationships (Fuligni and Eccles, 1993; Hallquist et al., 2015; Steinberg et al., 2006). While research has shown that the quality of the parent-child attachment relationship tends to influence the quality of peer and romantic partner attachment via internal working models that establish patterns of interpersonal relationships, it is also true that significant changes are made in the organization of attachment systems during adolescence and young adulthood such that the correlation between parent, peer and romantic partner attachment may diminish in some individuals (Gorrese and Ruggieri, 2012), resulting in attachment and mentalizing that are relationship-specific (O'Connor and Hirsch, 1999). Having questions asked for three attachment contexts meant that data analytic strategies had to take into account method factors influencing response patterns. It also raised the interesting question whether context-specific hypermentalizing probing was even necessary. Put differently, do factors that are comprised of shared variability of these three perspectives (parent, romantic partner, and closest friend) provide any incremental information beyond what would be obtained from the total score when responses across the three attachment context were summed? Additionally, it would be important to determine which of the three relationships contexts provided the most useful information about hypermentalizing compared to the others.

Third, given that hypermentalizing has been demonstrated in adolescent and adult populations using the MASC, and to facilitate studies in which the development of hypermentalizing can be tracked, it was desirable to develop items that could be used in adolescent and young adult samples.

To this end, we conducted two studies, each with a unique age cohort that covers the developmental period during which attachment begins to transition to include peer and romantic partners—that is, adolescence and young adulthood. Consistent with the World Health Organization's definition of the term “young people” to denote 10–24 year-olds, this developmental period extends from puberty (operationally defined as age 10–12 years), beyond traditional notions of adolescence (ending at age 18 years), to around 25 years of age (Dahl et al., 2018; Sawyer et al., 2018). Study 1 made use of a convenience sample of young adults in a college setting and its primary focus was twofold; first, to evaluate the factor structure of the newly developed measure, and second, to evaluate the association with the MASC as a criterion measure of hypermentalizing. Study 2 utilized an adolescent sample consisting of three groups: typically developing adolescents, adolescents with borderline pathology and adolescents with psychiatric problems (but no borderline pathology). The aim of Study 2 was to conduct a three-group comparison to give credence to the psychopathology roots of the hypermentalizing construct, and its particular significance for borderline personality pathology. For instance, individuals with social anxiety have also been shown to hypermentalize (Hezel and McNally, 2014; Washburn et al., 2016). A recent meta-analysis furthermore showed that hypermentalizing was not specific to BPD (McLaren et al., 2022). We hypothesized, however, that hypermentalizing would be more profoundly affected in borderline personality disorder compared to psychiatric caseness in general.

## Study 1: factor structure and associations with a criterion measure

Study 1 utilized a large college-based sample which afforded us the opportunity to explore two aims. First, we evaluated the factor structure of the measure given the increased variability of most constructs in non-clinical samples. In this study we were interested in determining the best model to explain covariance between items to ultimately justify the use of the parent-, romantic partner- and close friends versions of the measure, as well as the five subscales representing five underlying factors (an overconcern with the mental states of others; overinterpretation of others' mental states; inflexible certainty in own beliefs about others' mental states; acting impulsively on the assumed mental states of others; and second-guessing or over-interpretation of own mental states) of hypermentalizing. Second, we evaluated the associations with the gold standard measure of hypermentalizing, namely the MASC (Dziobek et al., 2006).

## Study 1 methods

### Participants

Data were collected from a sample of 745 undergraduate students at a large and racially and ethnically diverse university in an urban area in the southern region of the United States. Participants were recruited via a mass email advertising an online study to undergraduate students enrolled in at least one Psychology course. The recruitment email was sent from the Department of Psychology and participants self-selected to participate in this study by following a hyperlink to the University's online survey system. All responses were anonymous and identifiable only by a unique, randomly generated code. Inclusion criteria were English fluency and age between 18 and 25. There were no exclusion criteria. Participants were informed of the inclusion criteria in a cover letter and were instructed to self-exclude if the aforementioned criteria were not met. The sample included 586 women and 159 men (9 participants did not identify their gender). The mean age in this sample was 21.12 (*SD* = 2.19). The self-identified ethnoracial breakdown was 26.1% White/Not Hispanic, 14.7% Black, 31.5% Hispanic/Not White, 23.2% Asian or Pacific Islander, and 4% Multiracial or other (eight participants did not identify their ethnoracial background). This study was approved by the relevant Institutional Review Board and informed consent was provided. Participants completed questionnaires via a web-based program and were compensated with research credit.

### Measures

#### Hypermentalizing questionnaire (HMZQ)

The HMZQ consists of 26 items that are completed on a five-point Likert scale ranging from 0 (“not typical at all”) to 4 (“very typical). Respondents are asked to do the following: “*Below are 26 questions about the thoughts and feelings that are TYPICAL for you in interaction with your SIGNIFICANT OTHERS. We will ask the questions three times over. First, in the context of your relationships with your parents. Second, in the context of the relationship with your romantic partner (if you are currently not in a relationship, think back to your most recent relationship). And third, in the context of the relationship with your closest friend*.” Items were written to load onto one of five underlying factors: Overconcern for the mental state of others (OC), e.g., “I worry a lot about what my parents are thinking and feeling”; Overinterpretation of others' mental states (OI), e.g., “My parents often say I overinterpret interpersonal situations with them”; Inflexible certainty in beliefs about others' mental states (IC), e.g., “My feelings about what my parents are thinking are hardly ever wrong”; Acting impulsively on assumed mental states of others (IP), e.g., “I easily lose control in situations with my parents if my feelings get hurt”; Second-guessing/over-interpretation of own mental states (SG), e.g., “I often second-guess myself when interacting with my parents.” These components of hypermentalizing were derived from a thorough literate review of hypermentalizing (Sharp, 2014; Sharp and Vanwoerden, 2015). Items were written during a workshop of experts and piloted for among undergraduates in the first author's lab. Items were refined based on informal feedback.

#### Movie assessment for social cognition (MASC)

To explore criterion validity, participants completed the MASC (Dziobek et al., 2006), which is a computerized test for the assessment of mentalizing that approximates the demands of everyday life. Subjects watch a 15-min film about four characters getting together for a dinner party. Themes of each segment covered friendship and dating issues. During administration of the task, the film is stopped at 45 points and multiple-choice questions referring to the characters' mental states (feelings, thoughts, and intentions) are asked (e.g., “What is Betty feeling?”, “What is Cliff thinking?”). All items answered correctly are summed for a total score with higher scores indicating higher mentalizing capacity. The three incorrect responses are categorized as representing hypermentalizing, undermentalizing, or no mentalizing; counts of each of these incorrect responses make up the subscales of maladaptive mentalizing. The MASC is a reliable instrument that has proven sensitive in detecting subtle mindreading difficulties in adults of normal IQ (Dziobek et al., 2006) and in adolescents (Sharp et al., 2011).

### Data analytic strategy

To investigate the aims of study 1, we examined (1) competing factor models to determine which model or models were a reasonable fit to the data using four competing factor models described below, (2) if HMZQ trait factors accounted for unique criterion validity beyond total scores of the three types of relationships (parent close friend, and romantic partner) in higherarchical regression models, and (3) convergent and discriminant validity patterns between HMZQ trait factors, HMZQ total scores, and MASC dimensions, by examining the zero-order correlations between HMZQ factors/total scores and MASC dimensions.

To investigate the factor structure of the newly developed HMZQ, we investigated four competing models. All four models were non-nested so we relied on traditional fit indices: Comparative Fit Index (CFI), Tucker-Lewis Index (TLI) and Root Mean Square Error of Approximation (RMSEA). Item variance accounted to evaluate the best fitting model. The four competing models were (1) a single trait model with all items loading onto a hypermentalizing factor, (2) a single trait multimethod model with all items loading onto a hypermentalizing factor and the respective items loading onto parent, romantic partner, and close friend method factors, (3) a multi trait model with items loading onto OC, OI, IF, IP, and SG factor, and (4) a multi trait multimethod model with all items loading OC, OI, IF, IP, and SG trait factors and the respective items loading onto parent, romantic partner, and close friend method factors.

## Study 1 results

### Factor structure of the HMZQ

Model fit statistics are presented in Table 1.

**Table 1:** Hypermentalizing factor model fit indices.

**Models tested**	**CFI**	**TLI**	**RMSEA**
Model 1: single trait Unidimensional hypermentalizing trait with no modeling of the attachment context	0.761	0.754	0.085
Model 2: single trait, multi-method Unidimensional hypermentalizing trait with modeling of the attachment context	0.946	0.943	0.041
Model 3: multi-trait Hypermentalizing components modeled with no modeling of the attachment context	0.764	0.757	0.085
Model 4: multi-trait, multi-method Hypermentalizing components model with modeling of attachment context	0.948	0.945	0.040

To interpret model fit statistics for RMSEA, values of 0.05 and lower are generally good, while those between 0.05 and 0.08 are acceptable. For CFI and TLI values close to or higher than 0.09 are general good.

As can be seen, models 1 and 3, which did not include method factors did not fit well, but both models 2 and 4 did fit well. Model 4, the multi-trait multi-method model fit the best, but it's improvement over model 2, the single trait multi-method model was only marginal. Thus, for reasons of parsimony, it could be argued that model 2 is the preferable model. However, we retained both models 2 and 4 in our investigation of the best predictor of performance on the MASC. Finally, we also investigated factor models containing only parent items, only close friend items, and only romantic partner items. However, these factor scores correlated at or above 0.94 with their respective total scores. Thus, because the factor scores are near replications of the total scores, we dropped these factors from our predictive models of the MASC as a practical consideration of field use of the instrument.

### Relations between HMZQ and MASC

#### Descriptive statistics

Descriptive statistics and intercorrelations between the four subscales of the MASC (hypermentalizing, hypomentalizing, no mentalizing, and correct mentalizing), the HMZQ trait factor, the five HMZQ trait factors from the five factor model, the HMZQ total score, the HMZQ parent total, the HMZQ romantic partner total, and the HMZQ close friend total can be seen in Table 2.

**Table 2:** Descriptive statistics.

**Obs**	**Variable**	**Mean**	**Std. Dev**.	**1**	**2**	**3**	**4**	**6**	**7**	**8**	**9**	**10**	**11**	**12**	**13**
1	MASCTotal	17.19	15.65	–	–	–	–	–	–	–	–	–	–	–	–
2	MASCExcessiveToM	4.00	4.24	0.48^**^	–	–	–	–	–	–	–	–	–	–	–
3	MASCLessToM	3.23	3.83	0.36^**^	0.69^**^	–	–	–	–	–	–	–	–	–	–
4	MASCNoToM	1.93	2.99	0.17^**^	0.61^**^	0.76^**^	–	–	–	–	–	–	–	–	–
6	Overconcern others (OC)	0.01	0.84	0.37^**^	−0.25^**^	−0.39^**^	−0.35^**^	–	–	–	–	–	–	–	–
7	Over interpretation others (OI)	0.01	0.88	0.36^**^	−0.26^**^	−0.36^**^	−0.34^**^	0.7^**^	–	–	–	–	–	–	–
8	Inflexible certainty others (IC)	0.00	0.88	0.37^**^	−0.26^**^	−0.35^**^	−0.33^**^	0.8^**^	0.76^**^	–	–	–	–	–	–
9	Acting impulsively (IP)	0.01	0.91	0.36^**^	−0.26^**^	−0.34^**^	−0.32^**^	0.64^**^	0.98^**^	0.77^**^	–	–	–	–	–
10	Second-guessing self (SG)	0.01	0.95	0.38^**^	−0.28^**^	−0.36^**^	−0.34^**^	0.74^**^	0.91^**^	0.92^**^	0.94^**^	–	–	–	–
11	Grand_Total_Hypermentalizing	122.15	49.03	−0.11^**^	0.14^**^	0.05	0.07	0.05	0.03	0.04	0.02	0.03	–	–	–
12	Total_HMZ_Parents	41.49	18.98	−0.11^**^	0.14^**^	0.02	0.07	0.09^*^	0.08^*^	0.1^**^	0.07	0.08^*^	0.88^**^	–	–
13	Total_HMZ_Romantic	46.10	18.44	0.02	0.03	−0.05	−0.03	0.21^**^	0.23^**^	0.2^**^	0.21^**^	0.21^**^	0.85^**^	0.63^**^	–
14	Total_HMZ_ClosestFriend	34.95	19.58	−0.25^**^	0.22^**^	0.18^**^	0.2^**^	−0.14^**^	−0.18^**^	−0.14^**^	−0.17^**^	−0.16^**^	0.87^**^	0.66^**^	0.59^**^

* < .05,

** < .01.

#### Predictors of MASC hypermentalizing

To evaluate criterion validity, we sought to investigate both which variables predicted hypermentalizing on the MASC and if the factors identified in our two factor models accounted for additional variance beyond the HMZQ total scores. As seen in Table 2, all variables except the romantic partner total score have significant zero order correlations with MASC hypermentalizing. From a purely descriptive standpoint, the magnitude of the single trait factor had the highest correlation with MASC hypermentalizing.

To investigate if the trait factors from the single and multi-trait models accounted for incremental variability in MASC hypermentalizing beyond the parent, romantic partner, and close friend total scores, we examined two hierarchical regression models (1) a model with three sets of variables with set 1 consisting of the parent total score component of the HMZQ, set 2 consisting of the romantic partner and close friend total scores from the HMZQ, and set 3 being comprised of the hypermentalizing trait factor from the single factor multi method model, and (2) a model with the same two first sets as the previous model but where set 3 consists of the five trait factors from the multi trait multi method model. We chose this set because of past research suggesting that the parental relationship was the most well established relationship of the three types of attachment context given its longer duration. Additionally, the factor scores were entered last to determine if complex modeling, something many practitioners may not undertake when collecting client data, improved variance accounted in MASC hypermentalizing. The results of these two hierarchical regressions can be seen in Table 3.

**Table 3:** Hierarchical regressions.

**Variable**	**b1**	**SE1**	**b2**	**SE2**	**b3**	**SE3**	***R* ^2^**
**Model 1**							
Parent HMZ total	0.02^*^	0.01	0.01	0.01	0.01	0.01	
Set 1							0.015
Romantic partner HMZ total			−0.03^*^	0.01	−0.02	0.01	
Close friend HMZ total			0.05^*^	0.01	0.03^*^	0.01	
Set 2							0.060
Hypermentalizing trait factor					0.91^*^	0.16	
Set 3							0.108
**Model 2**							
Parent HMZ total	0.02^*^	0.01	0.01	0.01	0.02	0.01	
Set 1							0.015
Romantic partner HMZ total			−0.03^*^	0.01	−0.02	0.01	
Close friend HMZ total			0.05^*^	0.01	0.03^*^	0.01	
Set 2							0.060
OC trait factor					−0.51	0.37	
OI trait factor					2.07	1.38	
IF trait factor					−0.96	0.76	
IP trait factor					−3.30	2.03	
SG trait factor					1.50	1.51	
Set 3							0.111

In both hierarchical models all predictor sets accounted for significant incremental variance accounted in MASC hypermentalizing. These results suggest that the hypermentalizing factors identified in the factor modeling do account for incremental validity in MASC mentalizing. It should be noted that both hierarchical models accounted for comparable variability in MASC hypermentalizing (a difference of only 0.003) and thus, these results provide further support for the utility of the single factor hypermentalizing trait factor.

#### Correlations with MASC subscales

Finally, we investigated the patterns of association for the HMZQ total scores for all four components of the MASC: hypermentalizing, hypomentalizing, no mentalizing and accurate mentalizing. To study patterns, we first examined the patterns of significance in the zero order correlations. We noted that the HMZQ trait factor not only correlated with MASC hypermentalizing, but also with hypomentalizing and no mentalizing. The same was true for the five trait factors, except in the opposite direction. As mentioned in the description of the MASC, scores are somewhat dependent on each other—the higher the scores on any incorrect response (hyper-, hypo-, no-) the lower the total correct on the MASC. Thus, given that the pattern of correlations between the both the single trait factor and the five trait factors with hypermentalizing, hypomentalizing, no mentalizing were consistent, the results suggest that the HMZQ trait factor is measuring incorrect mentalizing regardless of the error (excessive/no/less). Likewise, all five trait factors seem to be measuring accurate mentalizing. The close friend total score seemed to follow a similar pattern to the single HMZQ trait factor. The romantic partner total score was unrelated to any facet of the MASC. Finally, the parent total related to MASC hypermentalizing but not to hypo- or no mentalizing. Thus, from these results, only the parent total score seems to discriminate between MASC hypermentalizing and hypo- and no mentalizing.

## Discussion study 1

The aim of Study 1 was to evaluate whether the purported factor structure of the HMZQ is supported. To this end, we examined four factor models and factor scores were evaluated for their associations with a criterion measure of mentalizing (the MASC). Results of the factor analyses demonstrated support for both a single trait multimethod model with all items loading onto a hypermentalizing factor and the respective items loading onto parent, romantic partner, and close friend method factors, as well as a multi trait multimethod model with all items loading onto the OC, OI, IF, IP, and SG trait factors and the respective items loading onto parent, romantic partner, and close friend method factors. To further explore the utility of each of these models, they were evaluated in regression analyses with MASC hypermentalizing as dependent variable and the factor scores of both models as predictor variables. Results of the regression analyses showed that models with both the single trait factor, as well as the multiple traits accounted for significant incremental validity in MASC hypermentalizing beyond the parent, close friend, and romantic partner total scores. Additionally, the two models were comparable in explaining variability in MASC hypermentalizing suggesting the utility of the single factor hypermentalizing trait factor as most parsimonious. Evaluation of the associations between method and HMZQ factors and MASC subscales demonstrated that the HMZQ factors were correlated with all MASC subscales, not just the hypermentalizing subscale of the MCAS. In this sense, the HMZQ factors provide convergent validity for MASC hypermentalizing, but not discriminant validity with hypomentalizing, no mentalizing, and accurate mentalizing. As such, the HMZQ factors may be more reflective of accurate mentalizing and not specifically to the error of hypermentalizing. On the other hand, the parent total score did follow the expected pattern with regard to convergent and discriminant validity with the parent total score relating to negatively with the MASC total, positively with hypermentalizing, and not significantly relating to hypo or no mentalizing. In this sense, the parent total score of the measure appears to be best at discriminating between different forms of mentalizing, although we do note that the trait factors do account for significant incremental validity in terms of prediction of MASC hypermentalizing.

## Study 2: clinical utility of the HMZQ

The aim of Study 2 was to investigate clinical utility. To this end, Study 2 made use of a clinical sample of adolescents who were well-characterized psychiatrically to derive a borderline vs. non-borderline psychiatric group, as well as a sample of typically developing adolescents recruited from the community. We specifically chose BPD as a comparison because the concept of mentalizing as used in psychotherapy originated in the context of BPD (Fonagy, 1991) and hypermentalizing was identified as a potentially unique correlate of BPD in its early conceptualization (Sharp et al., 2011; Sharp, 2014). To assess BPD, we use an interview-based measure of BPD so as to reduce shared method variance between the psychopathology measure (BPD) and the new developed self-report measure of hypermentalizing.

## Study 2 methods

### Participants

Participants for Study 2 included 320 adolescents who were recruited from a psychiatric inpatient unit that serves individuals with severe behavioral and emotional disorders. Of these adolescents, 97 met full criteria for borderline personality disorder (BPD) as determined by clinical interview (the CI-BPD; Zanarini et al., 2003). Additionally, 189 healthy controls were recruited from the community through schools and community programs. Inclusion criteria for both samples was sufficient proficiency in English to consent and complete the necessary assessments, and exclusion criteria were a diagnosis of schizophrenia or another psychotic disorder, an autism spectrum diagnosis, or an IQ of <70. A number of adolescents did not complete the hypermentalizing questionnaire (29 healthy controls, 68 psychiatric controls, and 27 with BPD); however, these adolescents did not differ from those who did complete the questionnaire in terms of age [healthy controls: *t*_(185)_ = −0.84, *p* = 0.40; psychiatric controls: *t*_(202)_ = −0.14, *p* = 0.89; BPD *t*_(95)_ = −0.50, *p* = 0.62] or gender [healthy controls: $\chi_{\left(1,188\right)}^{2}$ = 2.21, *p* = 0.14; psychiatric controls: $\chi_{\left(1,204\right)}^{2}$ = 0.04, *p* = 0.84; BPD $\chi_{\left(1,97\right)}^{2}$ = 0.33, *p* = 0.56]. Therefore, the final sample included 70 adolescents with BPD, 136 psychiatric controls, and 158 healthy controls.

The study was approved by the relevant Institutional Review Board and informed consent was provided. Adolescent inpatients were collectively assessed by doctoral-level clinical psychology students and/or trained clinical research assistants. Assessments were conducted independently and in private within the first 2 weeks following admission on the inpatient unit. Healthy controls were assessed by doctoral-level clinical psychology students and/or trained clinical research assistants in private assessment rooms at a local University or in schools where adolescents were recruited from.

### Measures

#### Hypermentalizing questionnaire (HMZQ)

The HMZQ as described in Study 1 was administered to all participants.

**The Childhood Interview for DSM-IV Borderline Personality Disorder** (CI-BPD; Zanarini et al., 2003) is a semi-structured diagnostic interview for use with children and adolescents. The CI-BPD assesses the nine DSM-IV criteria of BPD, which were unchanged in Section II of the DSM-5. Each criterion has a set of corresponding prompts used by the interviewer to investigate that criterion, from which they rate with a score of 0 (absent), 1 (probably present), or 2 (definitely present). Adolescents who meet five or more criteria at the 2-level meet diagnostic criteria for BPD. Additionally, a Total Score can be used as a dimensional measure of BPD features, which is a sum of scores for each of the 9 criteria (maximum score of 18). Excellent psychometric properties for this measure have been demonstrated in a sample of inpatient adolescents (Sharp et al., 2012) as well as high concordance (94%) between parents and adolescents on BPD diagnoses based on use of this measure (Wall et al., 2017). Interrater reliability was evaluated on 13% (*n* = 40) of inpatient cases using Cohen's Kappa statistic. There was strong agreement between the original interviewer and an independent rater of the recorded interview for the final BPD diagnosis (κ = 0.886, *p* < 0.001).

## Study 2 results

**Figure 1:**
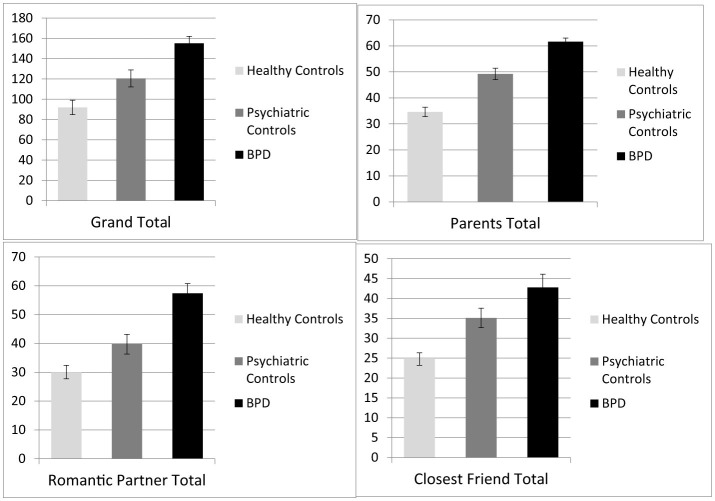
Group difference in HMZQ scores among adolescents meeting criteria for BPD, psychiatric controls, and healthy controls.

To determine the ability of the HMZQ to differentiate adolescents with BPD from those with other disorders and healthy controls, we compared the means of the various HMZQ scores across groups. Table 4 shows significant differences for all HMZQ scores with the largest effect size for the parent version of the measure. Tukey tests confirmed the superior performance of the parent version of the HMZQ by being the only HMZQ score that distinguished between all three subgroups. Specifically, comparisons of healthy controls vs. the BPD group (Tukey = −27.66; *p* < 0.001) and psychiatric controls vs. the BPD group (Tukey = −10.91; *p* = 0.04) showed significant differences for the parent version of the HMZQ. In contrast, only comparisons of healthy controls vs. the BPD group (Tukey = −26.20; *p* < 0.001), but not psychiatric controls vs. the BPD group (Tukey = −12.04; *p* = 0.21) showed significant differences for the romantic partner version of the HMZQ. Similarly, only comparisons of healthy controls vs. the BPD group (Tukey = −14.76; *p* < 0.001), but not psychiatric controls vs. the BPD group (Tukey = −1.69; *p* = 0.98) showed significant differences for the romantic partner version of the HMZQ. Taken together, only the total score and parent version of the HMZQ seems to be effective in distinguishing personality pathology from other psychopathology in youth. These results are depicted in Figure 1.

**Table 4:** Sample characteristics and HMZQ performance by group.

	**BPD *n* = 70**	**Psychiatric Non-BPD *n* = 136**	**Healthy controls *n* = 158**	**Group comparisons**
**Demographics**				
% Female	82.9	47.4	69.2	χ^2^ = 14.09, *df* = 2,365, *p* = 0.001
*M* Age (*SD*)	15.37 (1.52)	15.37 (1.30)	15.46 (1.28)	*F* = 1.74, *df* = 2, *p* = 0.18
%White/Not Hispanic	78.7	85.7	7.6	χ^2^ = 222.75, *df* = 10,338, *p* < 0.001
% Black	1.6	1.7	18.4	
% Hispanic/Not White	8.2	4.2	39.9	
% Multiracial or other	9.8	5	1.9	
**Psychiatric comorbidity**				
% Depressive disorder	72.9	65.4		χ^2^ = 3.32, *df* = 1,193, *p* = 0.07
% Bipolar disorder	11.4	3.8		χ^2^ = 5.29, *df* = 1,193, *p* = 0.02
% Eating disorder	20	6.6		χ^2^ = 9.46, *df* = 1,193, *p* = 0.002
% Externalizing disorder	51.4	33.1		χ^2^ = 8.84, *df* = 1,193, *p* = 0.003
% Anxiety disorder	71.4	59.6		χ^2^ = 5.66, *df* = 1,193, *p* = 0.02
**HMZQ**				
Total score (*SD*)	154.38 (42.07)	120.80 (56.41)	107.23 (51.66)	*F* = 14.34, *df* = 2, *p* < 0.001
Parent version (*SD*)	60.58 (20.24)	48.60 (19.44)	38.41 (20.29)	*F* = 34.74, *df* = 2, *p* < 0.001
Romantic partner (*SD*)	55.75 (18.94)	40.48 (23.22)	34.05 (22.87)	*F* = 18.05, *df* = 2, *p* < 0.001
Close friend (*SD*)	43.28 (22.35)	34.67 (21.81)	28.43 (18.01)	*F* = 13.24, *df* = 2, *p* < 0.001

40% of psychiatric controls, 25% of BPD patients, and 43% of healthy controls did not complete the romantic partner version of the HMZQ. Therefore, when calculating the total score on the HMZQ, group sizes were reduced due to the missing data for the romantic partner version of the measure to BPD (n = 55), psychiatric controls (n = 95) and healthy controls (n = 91). Psychiatric comorbidity determined using the Computerized Diagnostic Interview for DSM-IV (C-DISC; Shaffer et al., 2000) conducted with clients based on diagnostic criteria being met in the past year. The C-DISC was not completed in the healthy control sample.

## Discussion of study 2

The main goal of Study 2 was to assess the ability of the HMZQ to distinguish between psychiatric and non-psychiatric populations, and to investigate the specificity of HMZQ to borderline pathology. While our findings suggest that all versions of the HMZQ were good at distinguishing between healthy controls and borderline patients, only the total score and the parent version of the measure distinguished between adolescents with psychiatric disorders without BPD (psychiatric controls) and adolescents with BPD. This means that the romantic partner and best friend versions of the HMZQ are not sensitive to differences in groups with different psychopathology, but are most likely only sensitive to psychiatric severity in general. Given the theoretical roots of mentalizing in personality development and personality pathology, as discussed earlier, a measure that is sensitive to differences between general psychopathology and personality pathology would be considered more valid and fit for purpose. Given the attachment roots of the mentalizing construct as defined within Fonagy et al.'s (2002) model, it is perhaps not surprising that it is mentalizing in the context of the original attachment relationship—that is, the one with parents, that provide the best context for the assessment of hypermentalizing.

## Overall discussion

Hypermentalizing is a relatively new construct which has resonated with clinicians and researchers who routinely work with borderline personality disorder (Bo et al., 2015). While clinicians have often recognized the tendency in their patients to hypermentalize, until recently there had not been an empirically grounded construct available to describe or assess this tendency. For instance, psychodynamic object relation therapies have used the term “projective identification” to refer to a process akin to hypermentalizing. Projective identification was introduced by Melanie Klein and is broadly defined as the process whereby in a close relationship (e.g., often an attachment relationship or a relationship between a therapist and patient), parts of the self may in unconscious fantasy be thought of as being forced into the other person (Casement, 1990). Projective identification serves an important defensive function for the individual. Specifically, feelings which cannot be consciously accessed are defensively projected into another person in order *to evoke* the thoughts or feelings projected (Jacobs, 2006). Hypermentalizing is also evident in cognitive-behavioral writing in the form of “mindreading errors” (e.g., Burns, 1980) defined as making negative interpretations even though there is no definite fact that convincingly support the conclusion; for example, one arbitrarily concludes that someone is reacting negatively to one and one does not bother to check this out. The advantage of the hypermentalizing construct is that it is tied to a particular experimental task (the Movie Assessment for Social Cognition; MASC) and is empirically and conceptually grounded in the social-cognitive literature with associated clinical, behavioral, cognitive, and neurobiological correlates (e.g., Badcock, 2011; Franzen et al., 2011; Gambin et al., 2015; Langdon and Brock, 2008; Langdon and Coltheart, 1999; Sharp and Vanwoerden, 2015). In our view, a particular attractive feature of the hypermentalizing construct is the carefully articulated formulation of its developmental roots in attachment theory which provide additional conceptual coherence.

The aim of the current study was fully operationalize the construct of hypermentalizing and to facilitate the use of this construct in clinical and research settings in adolescents and young adults by developing and evaluating a self-report measure of hypermentalizing. To this end, we conducted two studies. The first made use of a college sample to evaluate the purported factor structure of the HMZQ and assess the validity of the derived factor structure by using the MASC as external validity measure. The second study explored the clinical utility of the newly developed measure in a sample of adolescents comprised of three groups: borderline, non-borderline psychiatric controls and healthy controls. Across the two studies, results provided preliminary support for the use of the parent version of the HMZQ in particular. In Study 1, while the factor structure of the single trait and multi-trait/multi-method factor models were both supported, and both models accounted for incremental validity in predicting hypermentalizing on the MASC beyond the parent, close friend, and romantic partner total scores, the parent total score was the only one that demonstrated both convergent and discriminant validity—positively correlating with hypermentalizing, negatively correlating with correct mentalizing and not correlating with hypomentalizing or undermentalizing. Study 2, in a different sample, confirmed the superiority of the parent version of the HMZQ in that it was only the HMZQ version that distinguished not only BPD from healthy controls but also BPD from psychiatric controls. Results of Study 1 also suggested little evidence in support of using the subscale scores for the purported factors (an overconcern with the mental states of others; overinterpretation of others' mental states; inflexible certainty in own beliefs about others' mental states; acting impulsively on the assumed mental states of others; and second-guessing or over-interpretation of own mental states) and the use of the total score of the parent version is recommended.

That the parent version of the HMZQ outperforms the romantic partner and close friend versions of the HMZQ in its association with hypermentalizing while at the same time performing almost as well as the single trait factor score, means that the 26 items comprising the parent version is the most parsimonious and effective way of capturing the latent construct of hypermentalizing. As discussed, the construct of mentalizing and hypermentalizing are both theoretically and empirically grounded in attachment; it is therefore no surprise that individual differences in hypermentalizing in the context of parental relationships appears to be sufficient for explaining variance in relevant outcomes. The outcomes in this study included experimentally defined hypermentalizing and borderline personality disorder, so it is left to be seen if the same would be true for other outcomes. Even so, attachment to parents developmentally precedes attachment to peers and is seen as the basis on which attachment to peers and romantic partners are built.

The current study has several limitations. First, construct validity of the HMZQ is partly based on group comparisons of scores on this measure leading to the possibility of response bias based on group characteristics (Millsap, 2011); therefore, future research should test measurement invariance of responses between clinical and non-clinical groups to determine whether the questionnaire functions in the same way across groups. Similarly, measurement invariance over time should be tested to determine whether development has an influence in responses to this questionnaire given the aim for this measure to have utility across adolescence and adulthood. Second, there were significant differences in demographics across samples in Study 2—specifically females were over-represented in both samples and there were significant socio-demographic differences between samples. While gender differences are in line with previous findings of higher prevalence of borderline personality disorder among females in clinical samples (Sansone and Sanson, 2011), ethnoracial differences across samples can partly be accounted for by the socioeconomic differences between the sample recruited from the community and the inpatient sample, which due to cost typically serves families with high incomes. Therefore, findings must be replicated in clinical samples that are more representative in terms of socioeconomic status and ethnicity/race. Findings should also be replicated in samples of older adults, and individuals from different cultural background, since the findings of the current study are generalizable to adolescent and young adults in the US context only. Finally, as with any self-report measure, there is the potential for response processes that are unrelated to the construct under consideration. However, concerns over the validity of self-report of mentalizing are somewhat mitigated by the fact that over the last two decades, a significant number of studies have been published suggesting strong psychometric properties for self-report measures of mentalizing in adolescents (e.g., Sharp et al., 2009; Ha et al., 2013; Duval et al., 2018; Lund et al., 2022; Sharp et al., 2021).

Notwithstanding these limitations, the current study introduces a self-report measure of hypermentalizing and provide preliminary evidence in support of its further validation using other approaches. The advantage of such a measure is that it can be used in clinical settings to assess the level of hypermentalizing errors associated with general psychopathology as well as personality pathology. Our results clearly showed significant differences for the total and parent hypermentalizing scores between healthy controls, those with psychiatric disorder (but no personality pathology) and those with personality disorder, with the latter group evidencing the highest levels of hypermentalizing. A relatively short and easy-to-administer measure of hypermentalizing facilitates the identification of reducing hypermentalizing in individuals with all forms of psychopathology, thereby expanding the hypermentalizing construct beyond its most common current application in personality pathology research and practice.

## Data Availability

The raw data supporting the conclusions of this article will be made available by the authors, without undue reservation.
